# PALLIATIVE CARE IN RESIDENTIAL CARE HOMES: IMPLEMENTATION CHALLENGES AND EARLY FINDINGS IN THREE CLINICAL TRIALS

**DOI:** 10.1093/geroni/igad104.1808

**Published:** 2023-12-21

**Authors:** Kathleen Unroe, Jenny van der Steen

## Abstract

This symposium will describe, compare and contrast three active clinical trials implementing palliative care models for people with dementia in residential care facilities and nursing homes. SPA-LTC is a pragmatic clinical trial in three Canadian provinces, providing training to palliative care teams and a process for triggered palliative care conferences. EMBED-Care, based in the United Kingdom, is testing a digital clinical support tool to improve integrated palliative care for people with dementia using holistic assessment and promoting shared decision-making. UPLIFT-AD is a multi-state U.S. based clinical trial, providing training for in-facility Palliative Care Leads and facilitating specialty palliative care clinician consults for people with moderate to advanced dementia. This symposium provides an opportunity to compare methodologic choices in study design, including intervention design, use of internal (care facility) based resources vs. external supports, and outcome assessment. Presenters will describe implications of these design choices for the implementation of the trial and for longer term sustainability. Implementation experience, including barriers and facilitators to palliative care programs for people with dementia, will be described.

